# Three-dimensional platinum nanoparticle-based bridges for ammonia gas sensing

**DOI:** 10.1038/s41598-021-91975-w

**Published:** 2021-06-15

**Authors:** Nishchay A. Isaac, Johannes Reiprich, Leslie Schlag, Pedro H. O. Moreira, Mostafa Baloochi, Vishal A. Raheja, Anna-Lena Hess, Luis F. Centeno, Gernot Ecke, Jörg Pezoldt, Heiko O. Jacobs

**Affiliations:** Fachgebiet Nanotechnologie, Technische Universität Ilmenau, Gustav-Kirchhoff-Strasse 1, 98693 Ilmenau, Germany

**Keywords:** Nanoparticles, Synthesis and processing, Nanowires, Sensors and biosensors, Design, synthesis and processing, Electrical and electronic engineering, Electronic properties and materials, Environmental, health and safety issues, Environmental impact

## Abstract

This study demonstrates the fabrication of self-aligning three-dimensional (3D) platinum bridges for ammonia gas sensing using gas-phase electrodeposition. This deposition scheme can guide charged nanoparticles to predetermined locations on a surface with sub-micrometer resolution. A shutter-free deposition is possible, preventing the use of additional steps for lift-off and improving material yield. This method uses a spark discharge-based platinum nanoparticle source in combination with sequentially biased surface electrodes and charged photoresist patterns on a glass substrate. In this way, the parallel growth of multiple sensing nodes, in this case 3D self-aligning nanoparticle-based bridges, is accomplished. An array containing 360 locally grown bridges made out of 5 nm platinum nanoparticles is fabricated. The high surface-to-volume ratio of the 3D bridge morphology enables fast response and room temperature operated sensing capabilities. The bridges are preconditioned for ~ 24 h in nitrogen gas before being used for performance testing, ensuring drift-free sensor performance. In this study, platinum bridges are demonstrated to detect ammonia (NH_3_) with concentrations between 1400 and 100 ppm. The sensing mechanism, response times, cross-sensitivity, selectivity, and sensor stability are discussed. The device showed a sensor response of ~ 4% at 100 ppm NH_3_ with a 70% response time of 8 min at room temperature.

## Introduction

Ammonia (NH_3_) is one of the most used industrial chemicals due to its widespread use as refrigeration gas, for fertilizers, in automobiles, plastics, explosives, textiles^1–4^. However, ammonia as a gas or vapor is toxic. Health effects include rapid skin/eye irritation and poisoning. As per American Industrial Hygiene Association (AIHA) emergency response planning guideline, exposure above 300 ppm of NH_3_ is dangerous to humans^5^. The Occupational Safety and Health Administration (OSHA) limits the NH_3_ 8-h total weighted average exposure to 25 ppm for human beings^6,7^. Various ammonia gas sensors have been developed which include—chemiresistive sensors^8–10^, optical sensors^11,12^, electrochemical sensors, surface acoustic wave sensors^13–15^, etc. Chemiresistive gas sensors sense a target gas concentration through a detectable change in their electrical resistance. They are extensively studied due to their compactness, low detection limit, fast response/recovery times, compatibility with integrated circuits, and cost-effectiveness. Those factors make them suitable for varied applications in environmental monitoring, automotive, industrial, and medical diagnostics^16–18^. However, most chemiresistive sensors operate at higher temperatures and require high power to maintain their gas sensing performance. Efforts are being made to produce room temperature operated sensors that are robust, energy-efficient, and show fast response to detect parts per million (ppm) concentration levels of ammonia gas.

Sensor performance can be measured in terms of: (i) Limit of detection—the ability of the sensor to detect the lowest possible target gas concentration, (ii) Selectivity—distinguish a particular target gas in a mixture of gases, (iii) Reversibility—the ability of a sensor to return to its original state after target gas exposure is removed^19^. Materials like transition/non-transition metal oxides, conductive polymers, and metals have been used for the development of such ammonia gas sensors. Both top-down and bottom-up approaches have been utilized to make solid-state, optical, electrochemical, or field-effect transistor sensors for ammonia gas detection^12,13^.

Gas-phase electrodeposition is one such bottom-up technique that can be used to fabricate such gas sensors based on metallic particles^20–22^. In simple terms, charged nanoparticles are produced using spark ablation. A carrier gas transports them to a conductive or semiconducting surface which is coated with a patterned resist. The openings in the resist define deposition locations. During the process, a steady-state electric charge will build up on the insulator, which directs charged nanoparticles from a carrier gas into the openings. Depending on the shape of the openings, nanostructures emerge and grow out of the openings. Various 3D-shaped nanostructures can be defined, including nanorods^23^, bridges^22^, and other 3D shapes^24^ composed out of nanoparticles.

Here, a study to fabricate conductometric NH_3_ gas sensors utilizing gas-phase electrodeposition as an alternative to conventional approaches is demonstrated. Gas-phase electrodeposition enables the deposition of an array of self-aligning 3D nanostructured bridges.

The deposition process is performed at room temperature at atmospheric pressure. As a proof-of-concept, we establish a sensor array to detect various levels of NH_3_. The bridges are deposited in such a way that each 3D bridge is connected in parallel to an external circuit providing a bias voltage for the gas sensing experiments. Instead of thin metallic films, point-to-point bridges made out of platinum (Pt) nanoparticles are used. Arrays containing 360 locally grown bridges are used to accomplish a stable sensor response. Different from the current state of the art, no external heating is required to operate the array. Due to the bridge microstructure, joule heating helps to obtain an increased sensor surface temperature, as shown with COMSOL Multiphysics simulation. The sensor performance is studied systematically with respect to selectivity, sensor stability, gas sensor response, and dependence on gas flows and humidity.

## Results and discussion

**Figure 1:**
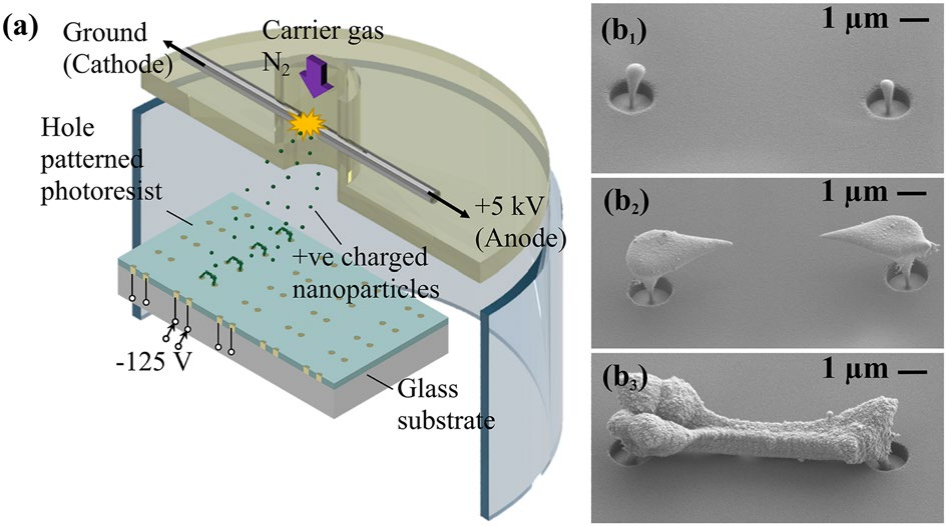
Gas-phase electrodeposition procedure schematics, bridge growth process, and nanobridge gas sensor test setup details. (**a**) Programmable gas-phase electrodeposition being carried out in a PMMA based reactor with a cylindrical cross-section. A spark discharge between platinum electrodes produces charged nanoparticles which are collected on a patterned substrate. During a particular time instant, platinum nanoparticles (green) are synthesized and collected on the biased domain pair until a bridge forms; all other domain pairs have a floating potential. This process is repeated 6 times to produce an array of Pt nanoparticle-based bridges on a single chip. (**b**) SEM images of a single bridge formation.

Gas-phase synthesis of nanomaterial is performed using spark ablation. Sparking is induced between two platinum electrodes with a gap of about 1 mm. Figure 1 introduces the gas-phase electrodeposition procedure. In brief, this process is based on the interplay of charged particles in the gas-phase with the following elements depicted in Fig. 1a: (i) charge dissipating electrodes, and (ii) patterned insulators. Inside the cylindrical PMMA reactor, the deposition substrate is shown. The substrate is made out of Pyrex glass and is coated with gold tracks (charge dissipating electrodes) which are connected to an external negative voltage source. This substrate is then coated with a patterned photoresist. The photoresist is an insulator and therefore, it will collect incident electrical charges during the process. This will increase its electrical potential up to a certain value. Charged species are produced during spark discharge and by diffusion and the carrier gas flow they are pushed onto the insulator surface where they accumulate. As more and more charges approach the insulator, the surface eventually starts to repel charged species with the same polarity (positive charges in the depicted case). The only places where the repelled charges can dissipate are circular openings in the photoresist. These openings provide access to the underlying gold tracks on the substrate. There, the species are electrically neutralized and deposited.

This gas-phase electrodeposition process is adapted to fabricate an array of nanobridges to enable a multi-nodal gas sensor. Figure 1b_1_,b_2_,b_3_ provide a closer look at the bridge formation process over time. The continuous collection of nanoparticles leads to the growth and eventual formation of nanoparticle-based bridges^22^. Due to localized charge dissipation the nanomaterial is funneled into the openings of the photoresist layer of the substrate. Hence, if the openings are placed in the x–y plane, nanomaterial growth will take place in the z-direction. As material grows out of the photoresist openings, nearest neighbor interaction guided by the electric field lines takes place^22^. Nanostructures growing out of the resist openings ‘see’ each other and connect to form 3D free-standing nanostructures. As an example, the depicted test substrate containes electrically independent domain pairs. The nearest-neighbor interaction is visible over this distance of several micrometers. In the depicted experiment, we used an identical 150-s-long nanoparticle gas-phase electrodeposition cycle on individual domain pairs.

**Figure 2:**
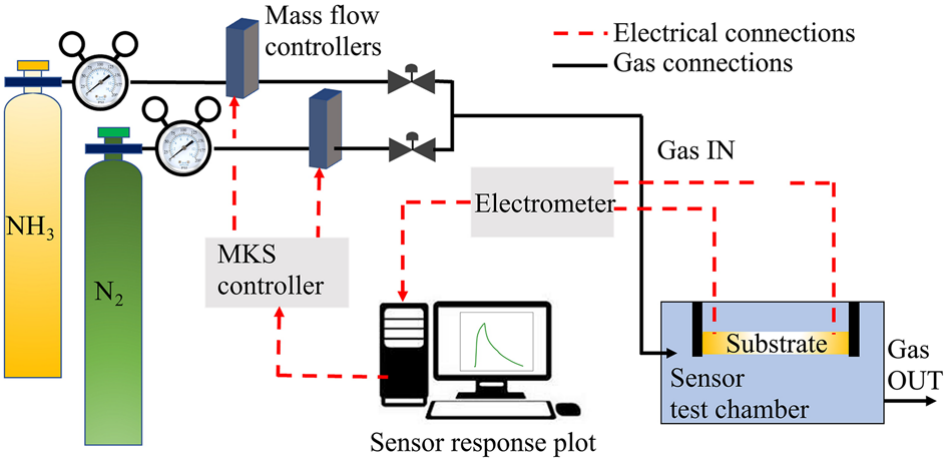
Schematic illustration of the sensor measurement set-up. Ammonia gas is diluted with Nitrogen 6.0 to concentrations of up to 100 ppm. During gas flow through the sensing chamber, the subsequent change in the electrical resistance of a bridge for a given externally applied voltage is recorded. The real-time performance is plotted on a computer with the help of a LabVIEW program.

Once the sensor chip is fabricated, it is installed in a separate sensing chamber with gas connections and electrical connections to the chip as shown in Fig. 2. A dilution system using two mass flow controllers provides target gas concentrations ranging from 1400 to 100 ppm. Nitrogen 6.0 (humidity-free) provided by Air Liquide is used as a background gas. To measure the resistance of the nanoparticle-based bridges, a bias voltage of 1.5 V is applied using an electrometer and the current is measured in real-time to calculate the electrical resistance using Ohm’s law. The room temperature and relative humidity inside the sensing chamber are maintained and logged in real-time using a digital thermometer-hygrometer-barometer (PCE-THB 40). The mass flow control and electrical measurements are integrated into a LabVIEW program programmed in-house which plots the gas sensor performance data in real-time. The bridges show ohmic behavior for electrical resistance calculations.

**Figure 3:**
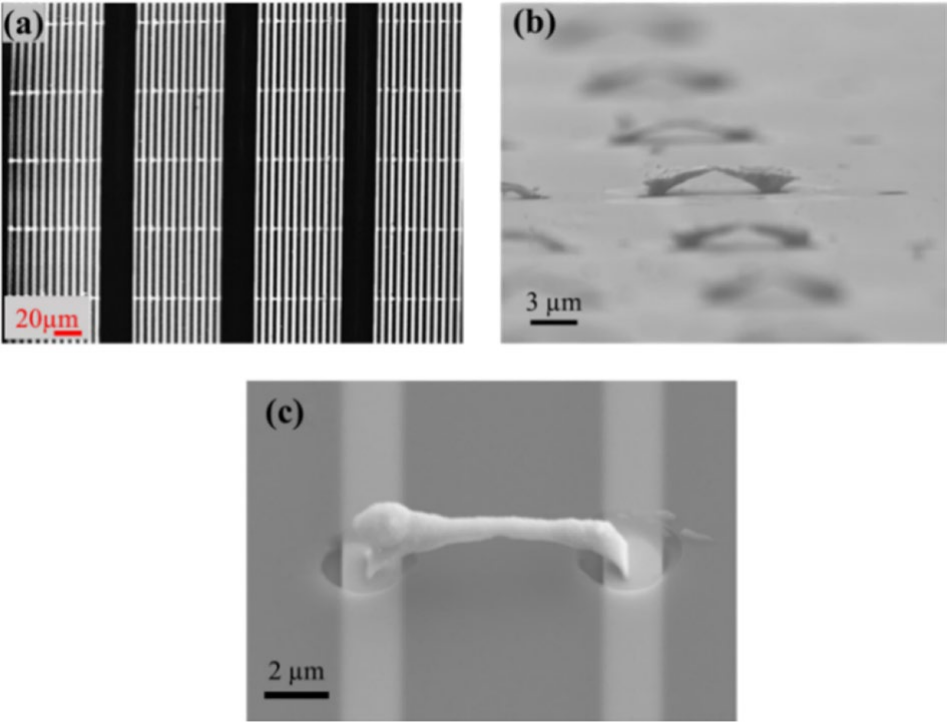
Nanobridge-based sensor array with multiple sensing nodes—fabrication overview. (**a**) A patterned photoresist with 2 µm openings is aligned to independent gold domain pairs. A negative bias of − 125 V is applied to the gold domains and 360 bridges are deposited in parallel. (**b**) While the openings are places in the x–y plane, the bridges grow out of the openings in the z-direction as shown in the SEM image with a 5-degree tilt. Such sensing nodes grown adjacent to each other cover the entire sensing area of the chip. (**c**) A single sensing bridge with its nanoparticulate sensing surface. All bridges are connected in parallel to the external circuit providing a bias voltage of 1.5 V.

Figure 3a illustrates the sensor layout and the corresponding scanning electron microscopy (SEM) images of the sensor array and a single Pt nanoparticle sensor. Multiple platinum bridges are deposited in a meander with 6 pairs of independent gold domains (Supplementary Information S1). In the presented layout, two neighboring electrodes form a domain pair. During a certain time, a domain pair is switched ON (biased) and nanomaterial is synthesized and collected on this electrode until all bridges growing out of the photoresist openings are formed. All other domain pairs have a floating potential during this step.

This process is repeated six times to produce an array of nanoparticle-based bridges on a single chip. The depicted bridges span a 9 µm distance and the bridge formation process takes 150 s. Two adjacent bridges are separated by 8 µm. This shows that a high density of nanomaterial devices can be replicated over large areas paving the way for multipurpose chips in the future. The fabricated chip provides room for a total number of 360 nanobridge-based gas sensing sites. The bridges grow out of the substrate from the patterned holes as can be seen in Fig. 3b.

The deposition of Pt nanoparticles leads to a bridge formation with a high surface-to-volume ratio which is required for low concentration NH_3_ sensing. An individual sensing node composed out of Pt nanoparticles is shown in the close-up SEM image (Fig. 3c). The underlying gold metal tracks are also visible. They provide charge dissipation to the incident charged nanoparticles during the bridge formation. The gas sensing area of the substrate for the experiment measures 1600 µm × 600 µm.

### Nanoparticle sensor material characterization

As mentioned, the particles are produced using spark discharge-based erosion of Pt electrodes. The process produces small (< 10 nm) particles with low coagulation^25,26^ and the particle size can further be reduced using a carrier gas to increase the quenching rate^22,27^.

**Figure 4:**
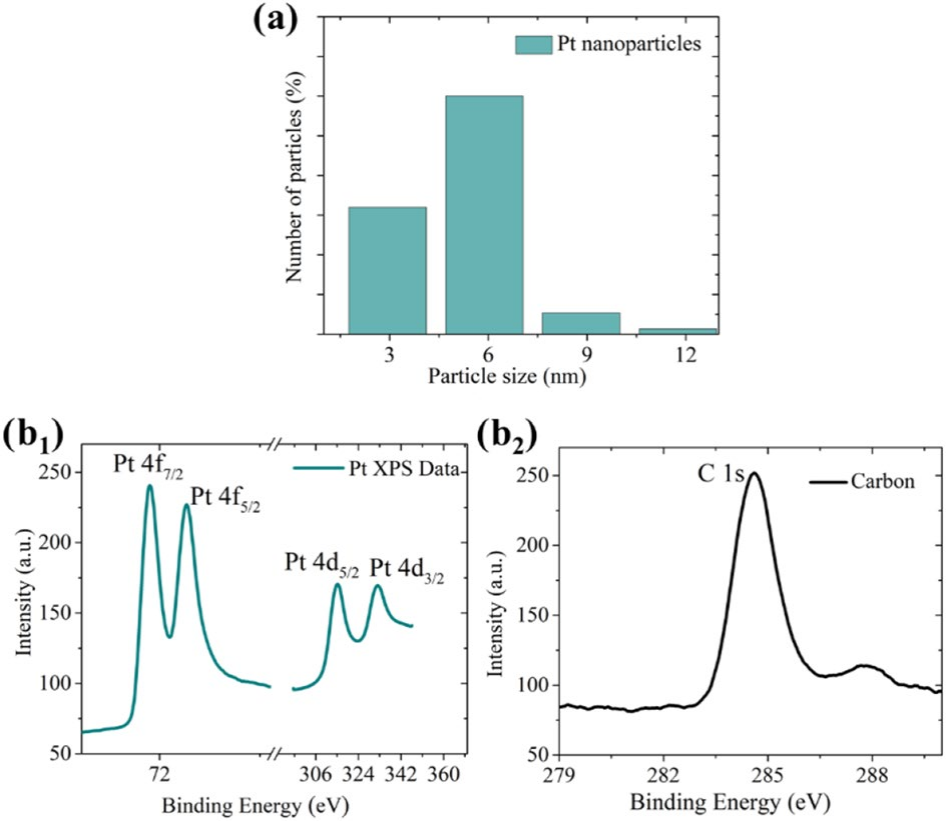
Particle size distribution and material characterization studies. At 6 slm gas flow through the spark discharge, primary Pt particles are collected on a Copper TEM grid and observed under a STEM Microscope. A total number of 75 particles are studied for this statistics. (**a**) Pt nanoparticles form a log-normal distribution with an average particle size of 5.3 nm. (**b**) Nanomaterial is deposited onto Si (111) substrates to perform XPS measurements using a surface analytics chamber with a monochromated Al Kα source. Pt 4f and Pt 4d XPS peaks (**b**_**1**_) are calibrated with adventitious carbon C–C bonds at 284.8 eV as shown in (**b**_**2**_).

At 6 standard liters per minute (slm) N_2_, the average particle size is ~ 5 nm for the produced Pt nanoparticles as shown in Fig. 4a_1_. The values were determined by measuring the size of Pt nanoparticles deposited on TEM grids using scanning transmission electron microscopy (STEM) (Supplementary Information S2). The average particle size of 5.3 nm and standard deviation (σ) of 2.1 is calculated from the observed log-normal distribution. Pt is a noble metal that is not prone to oxidation. In short, Pt remains pure throughout the processing sequence from the synthesis to the particle formation, localized deposition, bridge formation process, and thereafter. To confirm these statements, we analyzed the deposited particles using X-ray photoelectron spectroscopy (XPS).

Figure 4b_1_ shows the material characterization by XPS. In this investigation, calibration was made using binding energy for C1s peak, (C–C bonds at 284.8 eV) (Fig. 4b_2_), which is used in literature for inorganic surfaces exposed to air^28–30^. This is the basis for indexing other peaks for the nanobridge material. The doublet peaks are positioned as anticipated, specifically, the peaks of Pt 4d, are positioned at 314.6 eV (Pt 4d_5/2_), 332 eV (Pt 4d_3/2_), and the peaks of Pt 4f are positioned at 71.1 eV (Pt 4f_7/2_) and 74.4 eV (Pt 4f_5/2_). The slight shifts (0.4 eV) in the values of Pt 4d_5/2_ and Pt 4f_5/2_ are known to be related to grain size effects from literature^31,32^.

### Nanoparticle gas sensor characterization

**Figure 5:**
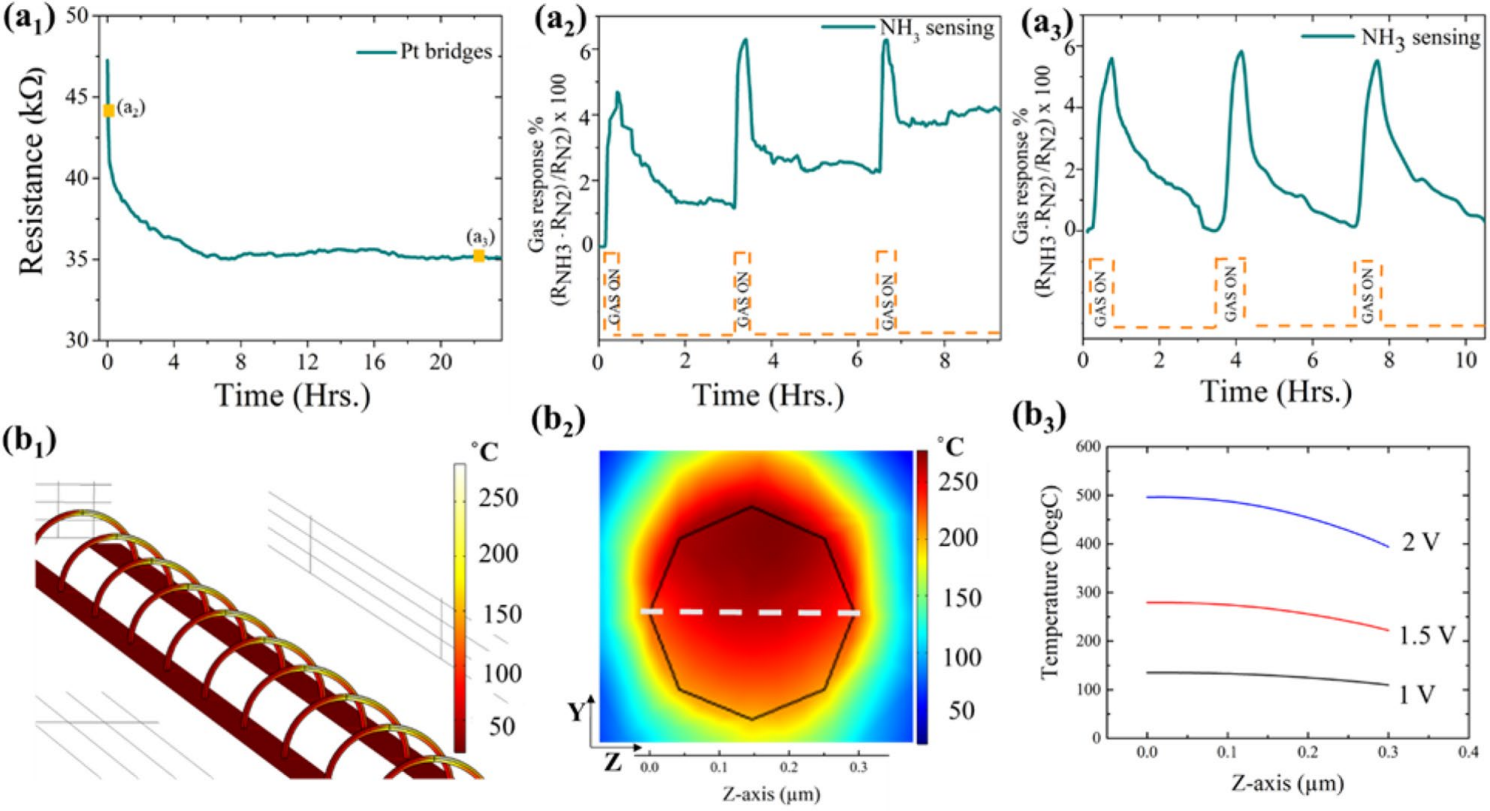
Gas sensor preconditioning and Joule heating simulations. Gas-sensitive bridges need to be placed in a constant flow of background gas for ~ 24 h to stabilize the gas sensor response. (**a**_**1**_) Pt sensing bridges decrease their overall bridge resistance during the preconditioning cycle. After this step, the bridge resistance is stable over time and hence is expected to reach stationary state with ambient conditions of temperature and gas species. If the NH_3_ response test starts without preconditioning, we obtain a sensor drift as shown in (**a**_**2**_), which decreased by over 80% after stabilization in (**a**_**3**_). (**b**) COMSOL Multiphysics simulations of the temperature of Pt nanoparticle-based bridges due to Joule heating at an external bias of 1 V, 1.5 V, and 2 V in 1 slm N_2_ flow are shown. It is assumed that the array of bridges (**b**_**1**_) is composed out of solid Pt nanowires with a 0.3 µm cross-section which is represented by a black polygon in (**b**_**2**_) where the temperature profile is shown as a heat map for an external bias of 1.5 V. This temperature profile is plotted as a function of position (white dashed line corresponds to the z-axis) in (**b**_**3**_). The nanowire temperatures are higher than the room temperatures which is the assumed reason for the decrease in bridge resistance during the pre-conditioning step.

Each nanobridge sensor chip is operated at typically 1.5 V with a power consumption of 50 µW. In the literature, today’s gas sensors have a power consumption in the µW range, but research aims for nW power consumption in the future. The power consumption when compared to other low power ammonia sensors reported in literature^33–38^ showed that it is equivalent and three orders of magnitude lower than a commercial FIGARO ammonia sensor TGS826. The sensor performance for the gas sensors in this article is reported in terms of gas sensor response (*S*). In this article, it is expressed as the ratio of difference in response signal when the sensing material is exposed to the target gas analyte and background gas to the response signal in the presence of background gas in percentage,

$$S\; (\% ) = \frac{{R_{G} - R_{A} }}{{R_{A} }} \times 100$$

where R_G_ is the resistance in the presence of target gas, and R_A_ is the resistance in the presence of a background gas. The sensor bridges were kept in a constant flow of 1 slm of background gas (N_2_) for ~ 24 h before sensing measurements were made. Figure 5a_1_ shows the effect of preconditioning on the sensor performance of platinum bridges.

As shown in Fig. 5a_1_, the resistance of the Pt bridges continuously decreased from 47 to 35 kΩ during preconditioning till stabilization. This is essential for accurate gas sensor performance studies. As seen in Fig. 5a_2_,a_3_, a non-preconditioned sensor has a high degree of sensor drift which can be decreased by over 80% through preconditioning. Perhaps this is possible due to the following reasons. Firstly, the gas sensor has a sub-micrometer size (~ 0.3 µm diameter) with a nanoparticulate structure, which can be heated by Joule heating. On the other hand, the carrier gas flow will have a cooling effect on the bridge. The result of the two competing effects can cause a change to the microstructure of the bridge which manifests itself in the form of a change in electrical resistance. This combined effect on such microstructures is shown through a simplified COMSOL multiphysics simulation in Fig. 5b_1_. An array of nanoparticle-based bridges is modeled as 6 µm long nanowires made out of solid Pt with a cross-section diameter of 0.3 µm. This array is placed in a flow of 1 slm *N*_*2*_ gas and a bias voltage of 1.5 V is applied. The nanobridges show a non-uniform temperature distribution. However, the area of the bridge showing the maximum temperature is sliced and a heat map of the circular cross-section is shown in Fig. 5b_2_. A line plot along this cross-section shows the surface and the core temperature of the bridge. Under the given assumptions, it is higher than the room temperature (Fig. 5b_3_). At 1.5 V bias, the bridge surface temperature is determined as ~ 275 °C. Higher temperatures are calculated with increasing bias voltages*.*

Secondly, the gas sensor is affected by the adsorption–desorption of gaseous species in the sensing chamber. This can cause a change in the resistance of the bridge. Only when a steady state between the bridge and its surrounding gas species has been achieved, the resistance of the bridge becomes independent of time and gas sensing measurements can start.

**Figure 6:**
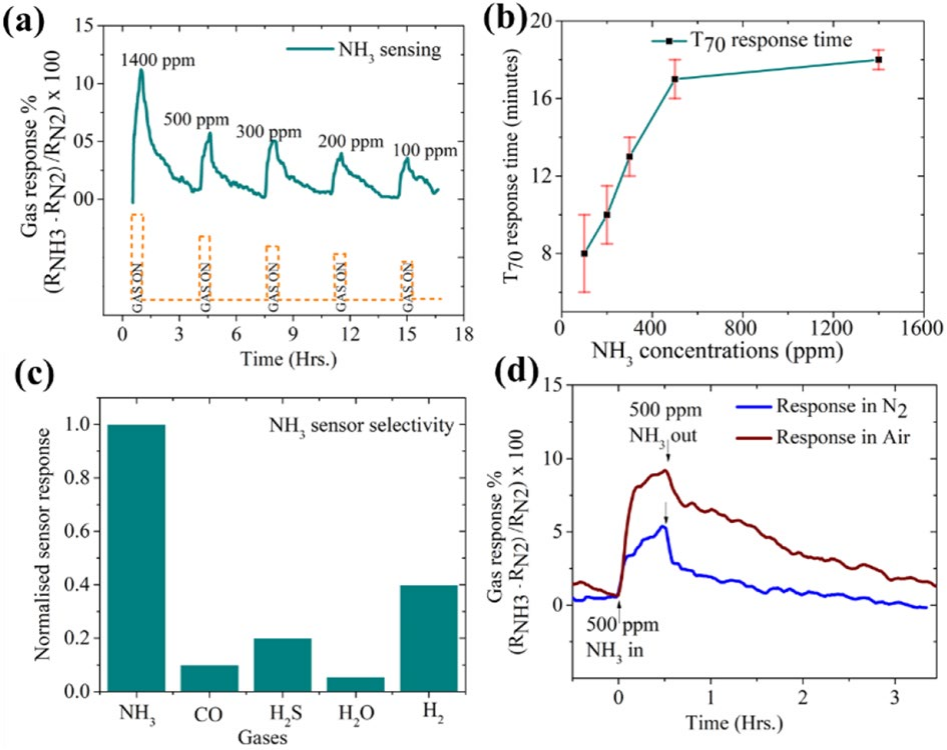
Gas sensor results. Bridges of Pt are employed for sensing varying concentrations of ammonia gas. Each NH_3_ concentration is exposed to the bridges for 30 min, and then they recover for 180 min in background of 6.0 N_2_ gas with no humidity. In (**a**) cycles of NH_3_ in N_2_ gas is introduced in gas sensor setup. In (**b**) 70% response times are plotted for different NH_3_ concentrations. Long-time sensor exposure to NH_3_ is undertaken to attain saturation value of resistance increase; the time taken to reach 70% of this saturation value is termed as T_70_ response time. (**c**) Normalized gas response of Pt bridges to various gases is plotted to show the extent of sensor cross sensitivity/selectivity. (**d**) Effect of background gas without humidity (N_2_ and air) is studied by observing the NH_3_ sensor response cycle. The gas sensor response is shown to improve owing to additional oxygen species helping in the NH_3_ adsorption and thus the sensing mechanism.

Figure 6 summarizes the NH_3_ sensing results with Pt nanobridges. First, we characterized the nanoparticle-based bridge array by exposing it to the NH_3_ concentrations that we would like to detect. Each gas concentration exposure duration was 30 min and the regeneration time in N_2_ 6.0 was 180 min. Five different NH_3_ concentrations down to 100 ppm were studied. Similar transient response curves were also examined for potential cross gases which can diminish the sensor selectivity. The sensor array was exposed to carbon monoxide (CO), hydrogen sulfide (H_2_S), humidity (H_2_O), and hydrogen (H_2_). The T_70_ response time is plotted as a function of the NH_3_ concentration. A 30 min exposure time was found to be insufficient to reach the saturation value of the gas response signal. To observe the saturation response signal value and to calculate the T_70_ response times, the gas sensor array was exposed for longer durations (~ 7 h) for each NH_3_ concentration. In this time, sensor response reaches 5% of the saturation value which was assumed to be stable for our sensor. However, the sensor required 20 h for a complete saturation (Supplementary Information S3). Based on this curve, the time to reach 70% of this saturation value was taken as response time. A short literature survey was performed to compare this proof-of-concept study with response times reported in the literature for ammonia gas sensors (Supplementary Information S4). The nanoparticle-based sensor in its current state has a slower response. Further studies are required to make this sensor comparable to the current state-of-the-art.

Figure 6a shows NH_3_ gas sensing by Pt nanoparticle-based bridges. The Pt nanoparticles cause catalytic dissociation of ammonia to nitrogen oxides as explained in literature by Kim et al.^39^

1 $$NH_{3\; adsorbed} + O_{adsorbed} \to NH_{2 \;adsorbed} + OH_{adsorbed}$$

2 $$NH_{2(adsorbed)} { } + O_{adsorbed} \to NH_{adsorbed} + OH_{adsorbed}$$

3 $$NH_{adsorbed} + O_{adsorbed} \to N_{adsorbed} + OH_{adsorbed}$$

4 $$NH_{adsorbed} + 2O_{adsorbed} \to NO_{adsorbed} + OH_{adsorbed}$$

5 $$NO_{adsorbed} + N_{adsorbed} \to N_{2} O_{(g)}$$

Oxygen species adsorbe on the Pt surfaces which was observed with XPS measurements (not shown), and agrees with the literature^40^. This plays a major role in ammonia adsorption and dissociation (Reactions 1–3). Ammonia gas dissociates on Pt surfaces by reacting with the adsorbed oxygen species giving NO (nitric oxide) and N_2_O (nitrous oxide)^39,41^ (Reactions 4, 5). The nitrogen oxides are electron-accepting in nature. They tend to decrease the free moving charge carriers (electrons) in Pt nanoparticles which results in an increased resistance.

In Fig. 6b, the response times as a function of NH_3_ concentration are plotted. An increase in the response time with increasing concentration can be explained through nanostructured bridge porosity. The 3D bridges presented in this manuscript have two types of porosity—a mesoporous surface and a microporous bulk which is shown in a SEM image on a scale of 100 nm (Supplementary Information S5). At low NH_3_ concentrations, the superficial mesoporous surface is easily accessible to most of the target gas molecules. Only a small fraction of gas molecules may reach the microporous bulk. On the other hand, at higher NH_3_ concentrations, the gas molecules can reach deep into the microporous bulk in the same amount of residence time due to the concentration gradient. However, this diffusion takes relatively longer times which, therefore, increases the response time. Similar trends have been observed in literature for ozone sensors^42^, other ammonia sensors^43,44^ and ethanol sensors^45^.

For any gas sensors, selectivity is a real issue because a myriad of gaseous species that are present in the ambient atmosphere could induce changes in the resistance of the bridges in a similar manner as the target gas. Sensor selectivity is also studied using various gas analytes which can potentially hinder the NH_3_ response. The gas sensor was exposed to 1000 ppm of NH_3_, CO, H_2_S, H_2,_ and 65% relative humidity (H_2_O) as shown in Fig. 6c. The value of water vapor is chosen to mimic the laboratory conditions. While Pt nanoparticle-based bridges showselectivity towards NH_3_, some undesired cross-sensitivity to carbon monoxide is observed. When CO adsorbs on the surface of the Pt bridge it acts as a weak electron acceptor. With this, CO is able to accept electrons from the Pt, which leads to an increased resistance in the bridge^46^. Similar behavior is observed with H_2_S since Pt nanoparticles are known to adsorb H_2_S molecules and S atoms^40^. Humidity has been a challenge for gas sensor development. A water molecule can dissociate as per the reaction: $H_{2}O \rightleftharpoons OH^{-} + H^{+}$. The hydroxyl ion (OH^−^) can interact with the nanomaterial forming a metal–OH dipole. As a result, the surface layer has few sites occupied by adsorbed OH^−^ ions, which cannot take part in NH_3_ detection. This can decrease the performance of the sensor. Pt bridges exposed with 65% relative humidity showed a cross-sensitivity of 5%. H_2_ adsorbs on platinum surfaces through the Langmuir–Hinshelwood mechanism, a catalytic mechanism for dissociative adsorption of H_2_ and O_2_ to produce –H and –OH groups on the gas-sensitive surface^47,48^.

Pt bridges show the highest cross-sensitivity to hydrogen with the signal being almost 40% that of NH_3_ response. Several approaches reported in the literature can be employed to enhance the gas sensor selectivity that include modulating the physical (e.g., grain size, operating temperature^49^), or the chemical (e.g., doping, surface functionalization^50^) properties of the gas-sensitive material.

An interesting observation is the effect of device response to the background gas. Ambient gases are known to cause electrical property changes in thin metallic films which have a similar surface as the bridges reported^51^. While all experiments in this proof-of-concept study have been carried out in nitrogen 6.0, the sensor response can be enhanced when humidity-free air is used as background gas as shown in Fig. 6d. These studies are carried out in dry conditions with no humidity. The sensing mechanism for Pt bridges is dependent on oxygen species adsorbed on the bridge surface. 20% O_2_ is available when humidity-free air is used as background gas. This increase in the availability of oxygen species promotes ammonia adsorption as well as dissociation to nitrogen oxides (Reactions 4, 5) which plays an active role in the gas sensor response^40^. However, a detailed study is required to ascertain the effect of using air (both under humid and dry conditions) for sensor response/recovery times, stability, selectivity, and sensitivity.

## Conclusions

A network of fully functional ammonia gas sensors made out of free-standing 3D nanobridges has been demonstrated which can sense up to 100 ppm of NH_3_. Gas-phase electrodeposition is used as a synthesis method. Nanostructured materials are synthesized in the gas phase through spark discharge. Spark discharge offers robust control on the nanoparticle morphology. With high gas flow rates, smaller nanoparticles (< 5 nm) can be synthesized^52^. Nanoparticle-based bridge growth occurs at charge dissipating contact points on a substrate and the formation of bridges is possible through nearest-neighbor coulombic interaction. A total number of 360 bridges (sensing nodes) are placed on a substrate and the gas sensor array performance is studied for Pt–NH_3_ combination. The gas sensors reported operate at room temperature and require preconditioning in background gas to decrease sensor drift making them robust for long durations of usage. Nanoparticle-based bridges with dimensions ranging to a few micrometers are expected to locally sinter due to Joule heating. This enables the operation of the nanobridge sensor at room temperature while locally the operating temperature of the bridges is higher. This means that the local operating temperature of the bridges can be easily controlled by changing the bias voltage as shown through preliminary COMSOL Multiphysics simulations.

The reported sensors show a 70% response time of ~ 8 min for 100 ppm of NH_3_. The response times increase with increasing gas concentrations. Further investigation of the bridge morphology and the sensor operation conditions are required to achieve faster response times and lower the limits of detection to sense trace quantities of NH_3_ gas in parts per billion. Such an in-depth investigation would pave the way for the future of 3D morphologies for room temperature and low power gas sensing.

## Materials and methods

### Nanomaterial synthesis

Gas-phase synthesis of nanomaterial is performed by spark ablation. Sparking is induced between two platinum electrodes (1 mm in diameter) with a gap of about 1 mm purchased from Advent Research Materials Ltd, UK. One electrode is at high voltage and the other one grounded, while there is a flow of carrier gas between them (6 slm N_2_). High voltage is provided using a block transformer from Gamma High Voltage Inc, USA. At the gas breakdown voltage, sparking initiates, locally heating the electrodes to form a metallic plume in the surrounding area. The spark frequency is maintained at 475 Hz. The metallic plume from the vaporized electrodes is quenched (to form nanoparticles) initially by radiation and adiabatic heating and subsequently by the flow of cold carrier gas between the electrodes.

### Substrate design

Pyrex glass wafers are coated with 10 nm chromium and 50 nm gold. This metal layer is patterned as metal tracks to form independent conductive domains on the wafer. Subsequently, AZ1505 photoresist (MicroChemicals GmbH) is spin-coated and patterned with circular openings towards the underlying metal tracks. The metal tracks are meander-shaped to accommodate an array of sensors in a small area, increasing the density of sensing nodes. The metal tracks end in pads that are connected to an external circuit which provides the negative bias voltage to collect positively charged nanoparticles produced by spark ablation. The total substrate size is 23 × 23 mm; however, the sensing area is 1600 × 600 µm^2^ located at the center of the chip. The chip is designed in such a way that it can be incorporated into an integrated circuit to form a plug-and-play device at a later stage.

## Supplementary Information

Supplementary Information.
