# Supplementary material for: Three-dimensional platinum nanoparticle-based bridges for ammonia gas sensing
Source: Sci Rep. 2021 Jun 15;11:12551. doi: 10.1038/s41598-021-91975-w (PMC8206144; doi:10.1038/s41598-021-91975-w)
Supplement: Supplementary file 1 — Supplementary Information. [file 41598_2021_91975_MOESM1_ESM.docx]

**Supplementary Information**

Three-Dimensional Platinum Nanoparticle-based Bridges for Ammonia Gas Sensing

Nishchay A. Isaac^1^, Johannes Reiprich^1^, Leslie Schlag^1^, Pedro H. O. Moreira^1^, Mostafa Baloochi^1^, Vishal A. Raheja^1^, Anna-Lena Hess^1^, Luis F. Centeno^1^, Gernot Ecke^1^, Jörg Pezoldt^1^, Heiko O. Jacobs^1*^

^1^Fachgebiet Nanotechnologie, Technische Universität Ilmenau, Gustav-Kirchhoff-Strasse 1 D-98693 Ilmenau, Germany

***Supplementary Information S1:***

The meander shape of the gold domains is explained schematically here.

| 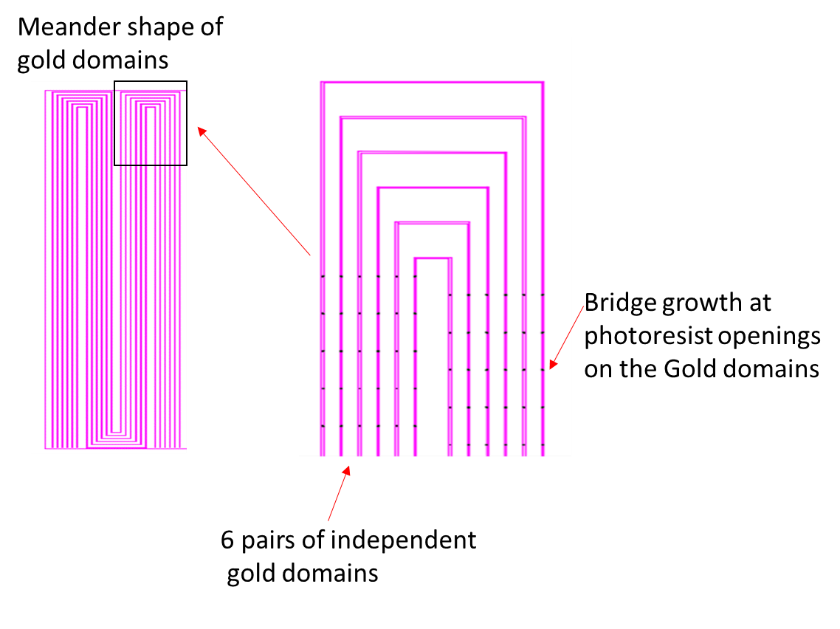 | ***Fig. S1***  *Meander shape of gold domains on the glass substrates. The meanders are coated with a layer of photoresist which is patterned with holes in it. The bridges grow independently on each gold domain pair when they are externally biased with a negative electrical voltage. The meanders are designed to provide a high density of ammonia sensors. A total number of 360 bridges are grown locally on the substrate.* |
| --- | --- |

***Supplementary Information S2:*** *Particle size distributions for Platinum nanoparticles collected on a copper grid.*

The particle size distribution calculations are as follows: a total number of 75 particles were analyzed for this. The particles were deposited on a copper grid and analyzed with a Zeiss Gemini 500 scanning electron microscope in transmission mode (STEM).


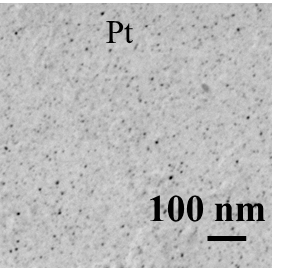


***Fig. S2.1*** *STEM micrograph of Pt nanoparticles collected on a Cu grid substrate to be analyzed for the particle size distribution. Randomly 75 particles were analyzed, and it was found that they have a log normal distribution.*

Average particle diameter: 5.3 nm

Standard deviation: 2.1 nm


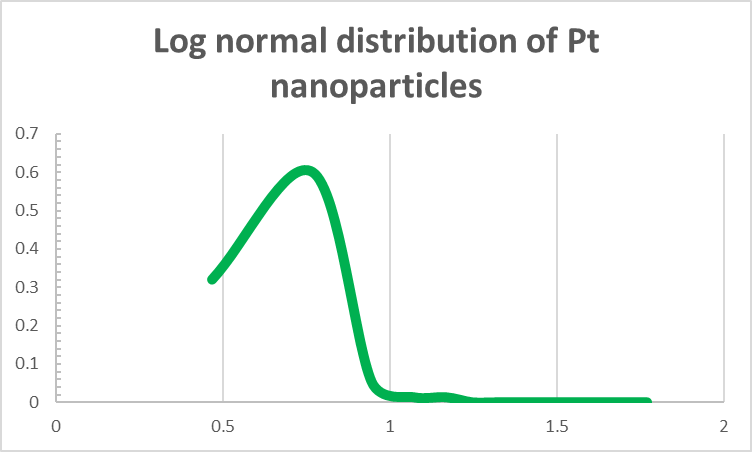


***Fig. S2.2*** *The log normal data calculated from the table is plotted*

***Supplementary Information S3:*** *Long-duration exposure of nanoparticle-based bridges to various ammonia concentrations for response time measurements.*

To calculate the response times, we make long exposures of different ammonia concentrations to the Platinum bridges. Once they reach saturation levels (within 5% of saturation is assumed as saturation value), we calculate the response times.


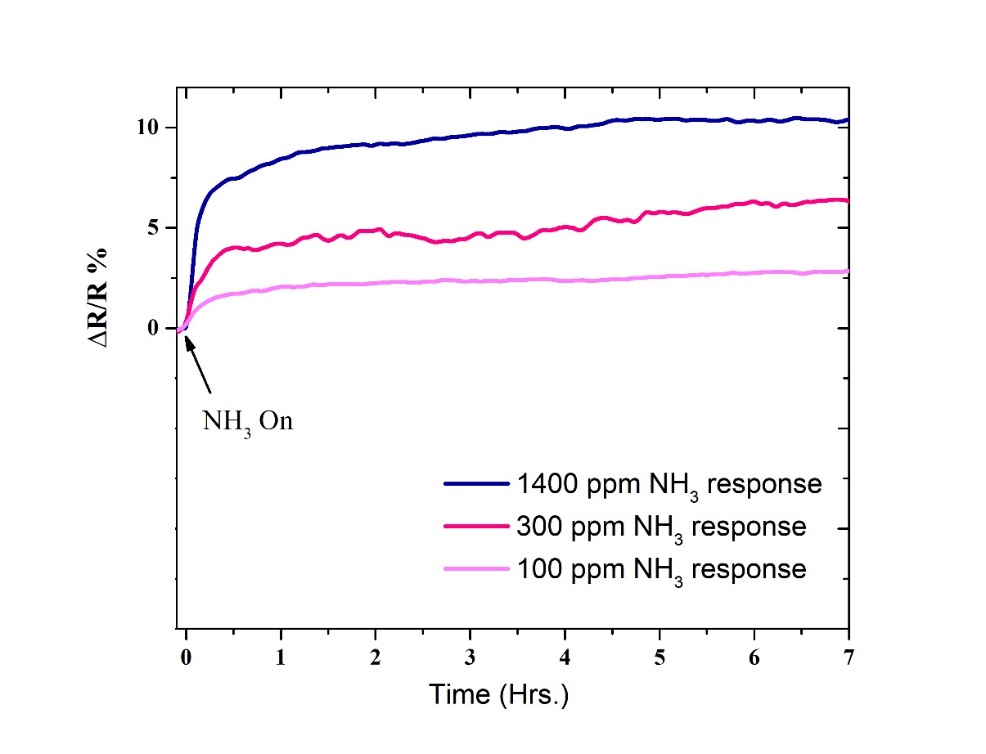


***Fig. S3.1*** *After 7 hours, the sensor response is within 5% of the saturation value and this was assumed to be stable for 70% response times calculations.*

One of the attempts to calculate the T_70_ response time is plotted as follows. The response times are calculated at 70% of the saturated value for the gas sensor.


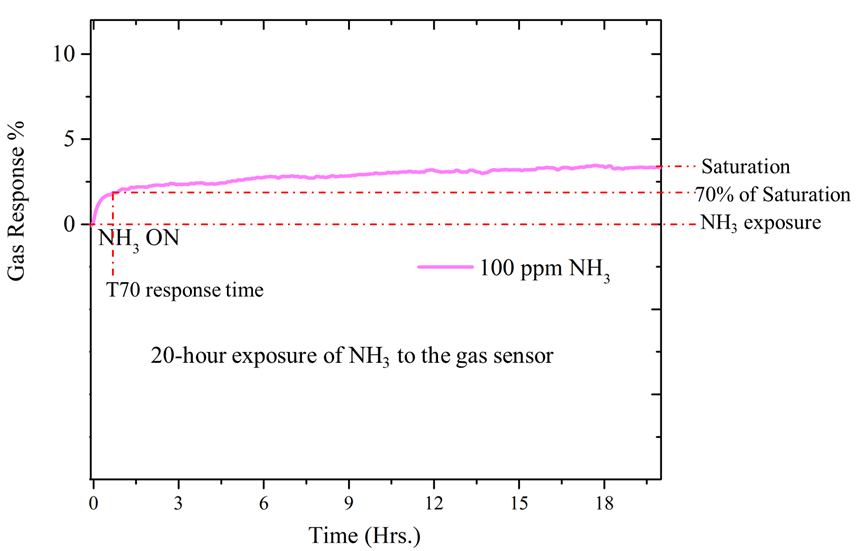


***Fig. S3.2*** *Gas sensor exposed to NH_3_ for 20 hours to reach a saturation value of the gas response. These saturation values for each concentration of NH_3_ are used to calculate the response times.*

***Supplementary Information S4:***

The sensors reported in literature use a 90% response time as compared to the 70% response times which are reported in this manuscript. Based on the gas response shown in Fig. S3.2, the 90% response time is calculated for the nanoparticle-based three-dimensional sensor and presented in Fig. S4.1. The 90% response time for the sensor in its current state is 198 minutes which is orders of magnitude slower than the current state-of-the-art (compare Table S4). Further studies are required to improve the sensorresponse kinetics

.**
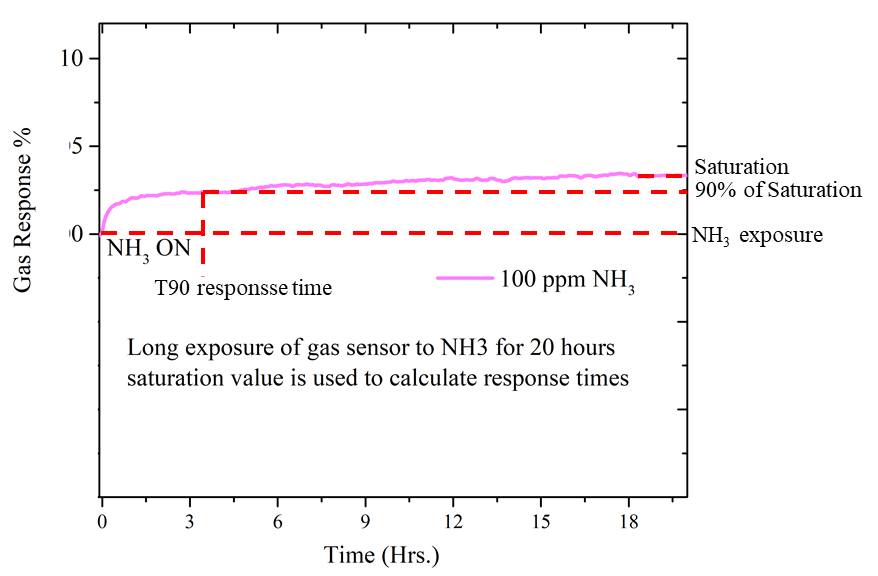
**

***Fig. S4.1*** *Gas sensor exposed to NH_3_ for 20 hours to reach a saturation value of the gas response. These saturation values for each concentration of NH_3_ are used to calculate the 90% response times to compare with other ammonia sensors reported in literature.*

***Table S4*** *Literature survey of response times for ammonia gas sensors and the corresponding concentrations for which these times are reported.*

| **Gas Detected** | **Response Time** | **Sensor Material** | **Concentration** | **Reference** |
| --- | --- | --- | --- | --- |
| Ammonia | 315 ± 20s –  structure A  200 ± 15 s –  structure B | Platinum nanomaterial | 10 ppm | ^1^ |
| Ammonia | 250 s  450 s | carbon nanofilms | 100 ppm  500 ppm | ^2^ |
| Ammonia | 100s | TiO_2_ nanocomposite | 100 ppm | ^3^ |
| Ammonia | 23 s | CaCO_3_ films | 1000 ppm | ^4^ |

***Supplementary Information S5:*** *Porosity*

The variation in the target gas response times can be explained with an SEM image of the bridge cross-section (obtained with Focused Ion Beam cut). At the scale of 100 nm, a microporous bulk can be seen.


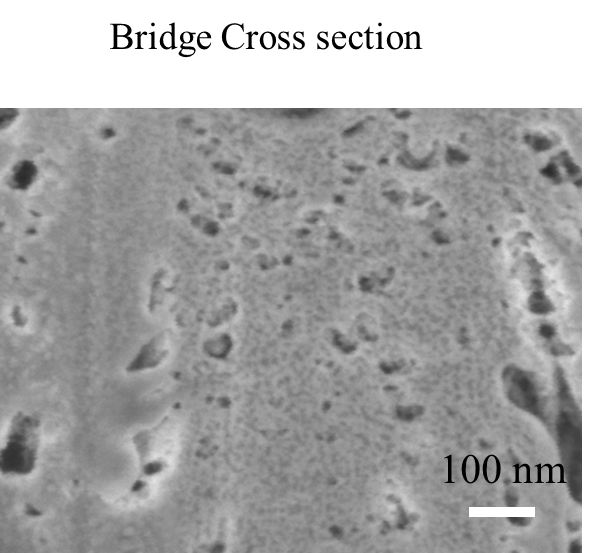


***Figure S5:*** *SEM micrograph of a Pt nanobridge cross-section with microporous structure*

REFERENCES

1. Hussain, G., Aldous, L. & Silvester, D. S. Preparation of platinum-based 'cauliflower microarrays’ for enhanced ammonia gas sensing. *Anal. Chim. Acta* **1048**, 12–21 (2019).

2. Bannov, A. G., Jašek, O., Prášek, J., Buršík, J. & Zajíčková, L. Enhanced Ammonia Adsorption on Directly Deposited Nanofibrous Carbon Films. *J. Sensors* **2018**, 7497619 (2018).

3. Zhu, C., Cheng, X., Dong, X. & Xu, Y. ming. Enhanced Sub-ppm NH3 Gas Sensing Performance of PANI/TiO2 Nanocomposites at Room Temperature. *Frontiers in Chemistry*  **6**, 493 (2018).

4. Sun, M., Li, Z., Gu, Y., Wu, S. & Wang, X. Room-temperature high-performance ammonia gas sensor based on hydroxyapatite film. *Mater. Res. Express* **6**, 106401 (2019).
